# Bis[*N*-(1-naphth­yl)ethyl­enediammonium] hexa­bromidoplumbate(II)

**DOI:** 10.1107/S1600536810008901

**Published:** 2010-03-17

**Authors:** Yao Chen, Ying-Ying Zheng, Gang Wu, Mang Wang, Hong-Zheng Chen, Hui Yang

**Affiliations:** a State Key Laboratory of Silicon Materials, Zhejiang University, Key Laboratory of Macromolecule Synthesis and Functionalization (Zhejiang University), Ministry of Education, Department of Material Science and Engineering, Zhejiang University, Hangzhou 310027, People’s Republic of China

## Abstract

The title compound, (C_12_H_16_N_2_)_2_[PbBr_6_], is an organic–inorganic salt, with two doubly protonated *N*-(1-naphth­yl)ethyl­enediammonium cations and one octa­hedral hexa­bromidoplumbate(II) anion. The Pb^II^ atom is located on a centre of inversion. The crystal structure consists of alternating inorganic and organic layers parallel to the *bc* plane. Face-to-face aromatic stacking inter­actions [centroid–centroid distance = 3.505 (5) Å] occur between parallel naphthalene systems in the organic layers, and N—H⋯Br hydrogen bonds between the cations and anions stabilize the crystal structure.

## Related literature

For the related structure bis­[*N*-(1-naphth­yl)ethyl­enedi­ammonium] hexa­iodido­plumbate(II), see: Zheng *et al.* (2007 ▶).
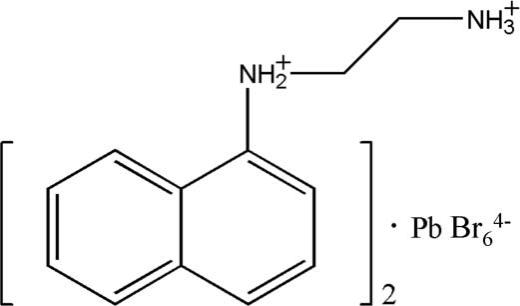


## Experimental

### 

#### Crystal data

(C_12_H_16_N_2_)_2_[PbBr_6_]
*M* *_r_* = 1063.19
Triclinic, 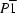

*a* = 8.1193 (4) Å
*b* = 8.5598 (4) Å
*c* = 12.4328 (6) Å
α = 80.4601 (13)°
β = 79.4756 (14)°
γ = 62.8592 (10)°
*V* = 752.63 (6) Å^3^
*Z* = 1
Mo *K*α radiation
μ = 13.59 mm^−1^
*T* = 296 K
0.39 × 0.33 × 0.20 mm

#### Data collection

Rigaku R-AXIS RAPID diffractometer
Absorption correction: multi-scan (*ABSCOR*; Higashi, 1995 ▶) *T*_min_ = 0.008, *T*_max_ = 0.066
6484 measured reflections
2938 independent reflections
2444 reflections with *I* > 2σ(*I*)
*R*_int_ = 0.092

#### Refinement

*R*[*F* ^2^ > 2σ(*F* ^2^)] = 0.067
*wR*(*F* ^2^) = 0.174
*S* = 1.00
2938 reflections
162 parameters
H-atom parameters constrained
Δρ_max_ = 3.73 e Å^−3^
Δρ_min_ = −3.27 e Å^−3^

### 

Data collection: *PROCESS-AUTO* (Rigaku, 2006 ▶); cell refinement: *PROCESS-AUTO*; data reduction: *CrystalStructure* (Rigaku, 2007 ▶); program(s) used to solve structure: *SHELXS97* (Sheldrick, 2008 ▶); program(s) used to refine structure: *SHELXL97* (Sheldrick, 2008 ▶); molecular graphics: *ORTEP-3 for Windows* (Farrugia, 1997 ▶); software used to prepare material for publication: *WinGX* (Farrugia, 1999 ▶).

## Supplementary Material

Crystal structure: contains datablocks global, I. DOI: 10.1107/S1600536810008901/xu2718sup1.cif

Structure factors: contains datablocks I. DOI: 10.1107/S1600536810008901/xu2718Isup2.hkl

Additional supplementary materials:  crystallographic information; 3D view; checkCIF report

## Figures and Tables

**Table 1:** Selected bond lengths (Å)

Pb1—Br1	3.0749 (8)
Pb1—Br2	2.9944 (10)
Pb1—Br3	3.0118 (10)

**Table 2:** Hydrogen-bond geometry (Å, °)

*D*—H⋯*A*	*D*—H	H⋯*A*	*D*⋯*A*	*D*—H⋯*A*
N1—H1*A*⋯Br1	0.90	2.48	3.364 (8)	168
N1—H1*B*⋯Br3^i^	0.90	2.89	3.618 (7)	139
N2—H2*B*⋯Br2^ii^	0.89	2.59	3.339 (9)	143

Symmetry codes: (i) 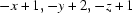; (ii) 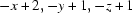.

**Fig. 1.**
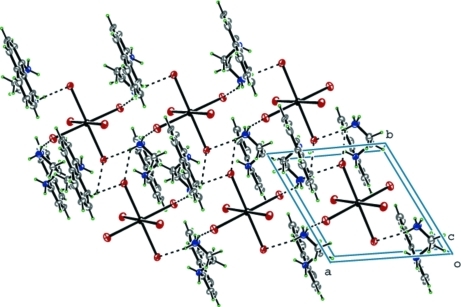
The molecular packing of the title compound viewed along the b axis.

**Fig. 2.**
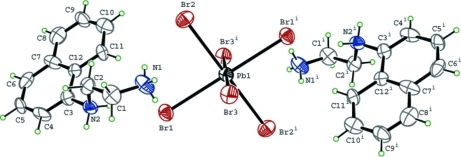
The structure of the title compound [symmetry code: (i) 1-x,1-y,1-z].

## supplementary crystallographic information

### Comment

The title compound is a organic-inorganic compound, its structure is similar to bis(N-(1-naphthyl)ethylenediammonium) hexaiodoplumbate(II) (Zheng *et al.*, 2007). The crystal structure is composed of alternating organic and inorganic sheets nearly parallel to the *bc* plane (Fig. 1). The Pb^II^ cation is located on an inversion center and coordinated by six Br^-^ anions with a distorted octahedral geometry (Fig. 2). The Pb—Br bond lengths (Table 1) are in the range form 2.9944 (10) to 3.0749 (8) Å. The face-to-face distance between adjacent parallel naphthalene ring systems is 3.505 Å, indicating aromatic π-π interaction. The N—H···Br hydrogen bonding is present in the crystal structure (Table 2).

### Experimental

The N-(1-naphthyl)ethylenediamine hydrobromide and PbBr_2_ are used as recieved. Concentrated hydrobromide and acetonitrile were degassed before using. All reactions were carried out under a nitrogen atmosphere. The title compound is prepared by a reaction of 0.1016 g N-(1-naphthyl)ethylenediamine hydrobromide with 0.0719 g PbBr_2_ in the mixture solution of 14.6 ml hydrobromide and 1.8 ml acetonitrile at 353 K. The resulting solution was kept at 353 K for 1 h and then slowly cooled down to room temperature. The single crystals were filtered off from the solution.

### Refinement

H atoms were placed in calculated positions with C—H = 0.93 (methylene), 0.97 (aromatic) and N—H = 0.89 or 0.90 Å, and included in the final cycles of the refinement in the riding-model approximation with U_iso_(H) = 1.2U_eq_(C,N) or 1.5U_eq_(N).

### Crystal data

**Table:**

(C_12_H_16_N_2_)_2_[PbBr_6_]	*Z* = 1
*M**_r_* = 1063.19	*F*(000) = 496
Triclinic, *P*1	*D*_x_ = 2.346 Mg m^−^^3^
Hall symbol: -P 1	Mo *K*α radiation, λ = 0.71073 Å
*a* = 8.1193 (4) Å	Cell parameters from 6259 reflections
*b* = 8.5598 (4) Å	θ = 3.0–27.4°
*c* = 12.4328 (6) Å	µ = 13.59 mm^−^^1^
α = 80.4601 (13)°	*T* = 296 K
β = 79.4756 (14)°	Chunk, colourless
γ = 62.8592 (10)°	0.39 × 0.33 × 0.20 mm
*V* = 752.63 (6) Å^3^	

### Data collection

**Table:**

Rigaku R-AXIS RAPID diffractometer	2938 independent reflections
Radiation source: rolling anode	2444 reflections with *I* > 2σ(*I*)
graphite	*R*_int_ = 0.092
Detector resolution: 10.00 pixels mm^-1^	θ_max_ = 26.0°, θ_min_ = 3.0°
ω scans	*h* = −9→10
Absorption correction: multi-scan (*ABSCOR*; Higashi, 1995)	*k* = −10→10
*T*_min_ = 0.008, *T*_max_ = 0.066	*l* = −15→15
6484 measured reflections	

### Refinement

**Table:**

Refinement on *F*^2^	Secondary atom site location: difference Fourier map
Least-squares matrix: full	Hydrogen site location: inferred from neighbouring sites
*R*[*F*^2^ > 2σ(*F*^2^)] = 0.067	H-atom parameters constrained
*wR*(*F*^2^) = 0.174	*w* = 1/[σ^2^(*F*_o_^2^) + (0.073*P*)^2^ + 3*P*] where *P* = (*F*_o_^2^ + 2*F*_c_^2^)/3
*S* = 1.00	(Δ/σ)_max_ = 0.002
2938 reflections	Δρ_max_ = 3.73 e Å^−^^3^
162 parameters	Δρ_min_ = −3.27 e Å^−^^3^
0 restraints	Extinction correction: *SHELXL97* (Sheldrick, 2008), Fc^*^=kFc[1+0.001xFc^2^λ^3^/sin(2θ)]^-1/4^
Primary atom site location: structure-invariant direct methods	Extinction coefficient: 0.0104 (12)

### Special details

**Table:**

Experimental. Spectroscopic analysis: IR (KBr, cm^-1^): 3008 (N—H asymmatric stretching), 2906 (N—H asymmatric stretching), 1573 (NH_2_ bending), 1142 (CH_2_ non-planar oscillating). Chemical analysis (calculated): C 27.09%, H 3.01%, N 5.27%; (found): C 27.12%, H 3.04%, N 5.23%.
Geometry. All e.s.d.'s (except the e.s.d. in the dihedral angle between two l.s. planes) are estimated using the full covariance matrix. The cell e.s.d.'s are taken into account individually in the estimation of e.s.d.'s in distances, angles and torsion angles; correlations between e.s.d.'s in cell parameters are only used when they are defined by crystal symmetry. An approximate (isotropic) treatment of cell e.s.d.'s is used for estimating e.s.d.'s involving l.s. planes.
Refinement. Refinement of F^2^ against ALL reflections. The weighted R-factor wR and goodness of fit S are based on F^2^, conventional R-factors R are based on F, with F set to zero for negative F^2^. The threshold expression of F^2^ > 2sigma(F^2^) is used only for calculating R-factors(gt) etc. and is not relevant to the choice of reflections for refinement. R-factors based on F^2^ are statistically about twice as large as those based on F, and R- factors based on ALL data will be even larger.

### Fractional atomic coordinates and isotropic or equivalent isotropic displacement parameters (Å^2^)

**Table:**

	*x*	*y*	*z*	*U*_iso_*/*U*_eq_	
Pb1	0.5000	0.5000	0.5000	0.03697 (12)	
Br3	0.69148 (13)	0.55171 (12)	0.66759 (8)	0.0503 (2)	
Br2	0.85539 (12)	0.36179 (13)	0.34710 (8)	0.0533 (3)	
Br1	0.44025 (12)	0.86889 (11)	0.39152 (8)	0.0482 (2)	
N2	0.8772 (11)	0.7533 (10)	0.4518 (7)	0.054 (2)	
H2A	0.7695	0.7973	0.4951	0.081*	
H2B	0.9659	0.6741	0.4912	0.081*	
H2C	0.8658	0.7018	0.3988	0.081*	
N1	0.7162 (9)	1.0520 (8)	0.2540 (6)	0.0418 (17)	
H1A	0.6464	0.9944	0.2816	0.050*	
H1B	0.6673	1.1530	0.2863	0.050*	
C1	0.7317 (10)	0.9604 (10)	0.0702 (7)	0.0371 (19)	
C6	0.7273 (11)	1.0045 (10)	−0.0447 (8)	0.043 (2)	
C10	0.7082 (12)	1.0940 (10)	0.1348 (8)	0.042 (2)	
C2	0.7567 (12)	0.7896 (10)	0.1148 (8)	0.042 (2)	
H2	0.7528	0.7596	0.1903	0.051*	
C11	0.9145 (12)	0.9392 (12)	0.2815 (8)	0.047 (2)	
H11A	0.9668	0.8306	0.2465	0.056*	
H11B	0.9895	1.0014	0.2513	0.056*	
C9	0.6906 (13)	1.2553 (10)	0.0898 (9)	0.050 (2)	
H9	0.6822	1.3366	0.1343	0.059*	
C5	0.7545 (13)	0.8761 (12)	−0.1131 (9)	0.051 (2)	
H5	0.7519	0.9045	−0.1885	0.061*	
C12	0.9269 (12)	0.8946 (12)	0.4031 (8)	0.051 (2)	
H12A	1.0534	0.8616	0.4159	0.061*	
H12B	0.8454	0.9995	0.4402	0.061*	
C8	0.6851 (12)	1.2998 (10)	−0.0233 (8)	0.049 (2)	
H8	0.6703	1.4115	−0.0540	0.058*	
C4	0.7839 (13)	0.7129 (12)	−0.0684 (9)	0.056 (3)	
H4	0.8025	0.6291	−0.1141	0.067*	
C7	0.7014 (12)	1.1792 (12)	−0.0885 (8)	0.046 (2)	
H7	0.6957	1.2109	−0.1635	0.055*	
C3	0.7876 (13)	0.6654 (11)	0.0432 (9)	0.052 (3)	
H3	0.8106	0.5506	0.0711	0.063*	

### Atomic displacement parameters (Å^2^)

**Table:**

	*U*^11^	*U*^22^	*U*^33^	*U*^12^	*U*^13^	*U*^23^
Pb1	0.03482 (19)	0.03828 (18)	0.0450 (3)	−0.02084 (16)	−0.00683 (18)	−0.00605 (16)
Br3	0.0562 (4)	0.0602 (4)	0.0498 (5)	−0.0361 (4)	−0.0112 (4)	−0.0079 (4)
Br2	0.0390 (4)	0.0651 (5)	0.0514 (6)	−0.0190 (4)	−0.0035 (4)	−0.0078 (4)
Br1	0.0429 (4)	0.0463 (4)	0.0596 (6)	−0.0249 (3)	−0.0068 (4)	0.0014 (4)
N2	0.051 (4)	0.055 (4)	0.062 (5)	−0.028 (3)	−0.020 (4)	0.007 (4)
N1	0.040 (3)	0.039 (3)	0.054 (4)	−0.022 (3)	−0.003 (3)	−0.014 (3)
C1	0.029 (3)	0.042 (3)	0.046 (5)	−0.018 (3)	−0.001 (3)	−0.011 (3)
C6	0.029 (3)	0.040 (3)	0.063 (6)	−0.015 (3)	−0.012 (4)	−0.008 (4)
C10	0.041 (4)	0.045 (4)	0.045 (5)	−0.024 (3)	−0.006 (4)	−0.003 (3)
C2	0.049 (4)	0.042 (3)	0.048 (5)	−0.029 (3)	−0.009 (4)	−0.001 (3)
C11	0.039 (4)	0.052 (4)	0.057 (6)	−0.026 (4)	−0.001 (4)	−0.008 (4)
C9	0.055 (5)	0.037 (3)	0.066 (6)	−0.028 (3)	−0.006 (5)	−0.009 (4)
C5	0.046 (4)	0.063 (5)	0.053 (6)	−0.030 (4)	−0.009 (4)	−0.008 (4)
C12	0.051 (4)	0.054 (4)	0.063 (6)	−0.032 (4)	−0.022 (4)	0.000 (4)
C8	0.047 (4)	0.038 (4)	0.063 (6)	−0.025 (3)	−0.002 (4)	0.002 (4)
C4	0.053 (5)	0.055 (4)	0.070 (7)	−0.025 (4)	−0.008 (5)	−0.025 (4)
C7	0.038 (4)	0.058 (4)	0.038 (5)	−0.022 (4)	0.000 (4)	0.004 (4)
C3	0.045 (4)	0.045 (4)	0.071 (7)	−0.020 (4)	−0.009 (5)	−0.011 (4)

### Geometric parameters (Å, °)

**Table:**

Pb1—Br1	3.0749 (8)	C10—C9	1.354 (12)
Pb1—Br1^i^	3.0749 (8)	C2—C3	1.405 (13)
Pb1—Br2	2.9944 (10)	C2—H2	0.9300
Pb1—Br2^i^	2.9944 (10)	C11—C12	1.506 (13)
Pb1—Br3^i^	3.0118 (10)	C11—H11A	0.9700
Pb1—Br3	3.0118 (10)	C11—H11B	0.9700
N2—C12	1.451 (12)	C9—C8	1.399 (14)
N2—H2A	0.8900	C9—H9	0.9300
N2—H2B	0.8900	C5—C4	1.343 (14)
N2—H2C	0.8900	C5—H5	0.9300
N1—C10	1.472 (12)	C12—H12A	0.9700
N1—C11	1.522 (10)	C12—H12B	0.9700
N1—H1A	0.9000	C8—C7	1.361 (14)
N1—H1B	0.9000	C8—H8	0.9300
C1—C2	1.410 (11)	C4—C3	1.382 (15)
C1—C6	1.419 (13)	C4—H4	0.9300
C1—C10	1.428 (12)	C7—H7	0.9300
C6—C5	1.414 (13)	C3—H3	0.9300
C6—C7	1.436 (13)		
			
Br2—Pb1—Br2^i^	180.0	C1—C10—N1	119.1 (7)
Br2—Pb1—Br3^i^	88.45 (3)	C3—C2—C1	118.9 (9)
Br2^i^—Pb1—Br3^i^	91.55 (3)	C3—C2—H2	120.6
Br2—Pb1—Br3	91.55 (3)	C1—C2—H2	120.6
Br2^i^—Pb1—Br3	88.45 (3)	C12—C11—N1	113.4 (7)
Br3^i^—Pb1—Br3	180.0	C12—C11—H11A	108.9
Br2—Pb1—Br1	86.43 (3)	N1—C11—H11A	108.9
Br2^i^—Pb1—Br1	93.57 (3)	C12—C11—H11B	108.9
Br3^i^—Pb1—Br1	92.47 (3)	N1—C11—H11B	108.9
Br3—Pb1—Br1	87.53 (3)	H11A—C11—H11B	107.7
Br2—Pb1—Br1^i^	93.57 (3)	C10—C9—C8	120.1 (9)
Br2^i^—Pb1—Br1^i^	86.43 (3)	C10—C9—H9	119.9
Br3^i^—Pb1—Br1^i^	87.53 (3)	C8—C9—H9	119.9
Br3—Pb1—Br1^i^	92.47 (3)	C4—C5—C6	119.6 (10)
Br1—Pb1—Br1^i^	180.0	C4—C5—H5	120.2
C12—N2—H2A	109.5	C6—C5—H5	120.2
C12—N2—H2B	109.5	N2—C12—C11	114.6 (8)
H2A—N2—H2B	109.5	N2—C12—H12A	108.6
C12—N2—H2C	109.5	C11—C12—H12A	108.6
H2A—N2—H2C	109.5	N2—C12—H12B	108.6
H2B—N2—H2C	109.5	C11—C12—H12B	108.6
C10—N1—C11	112.3 (7)	H12A—C12—H12B	107.6
C10—N1—H1A	109.2	C7—C8—C9	119.8 (8)
C11—N1—H1A	109.2	C7—C8—H8	120.1
C10—N1—H1B	109.2	C9—C8—H8	120.1
C11—N1—H1B	109.2	C5—C4—C3	122.1 (10)
H1A—N1—H1B	107.9	C5—C4—H4	118.9
C2—C1—C6	119.0 (8)	C3—C4—H4	118.9
C2—C1—C10	123.5 (8)	C8—C7—C6	121.8 (9)
C6—C1—C10	117.5 (7)	C8—C7—H7	119.1
C5—C6—C1	120.0 (8)	C6—C7—H7	119.1
C5—C6—C7	121.9 (9)	C4—C3—C2	120.4 (9)
C1—C6—C7	118.1 (8)	C4—C3—H3	119.8
C9—C10—C1	122.5 (9)	C2—C3—H3	119.8
C9—C10—N1	118.2 (8)		
			
C2—C1—C6—C5	−2.2 (12)	C1—C10—C9—C8	3.4 (14)
C10—C1—C6—C5	178.4 (8)	N1—C10—C9—C8	179.1 (8)
C2—C1—C6—C7	179.9 (8)	C1—C6—C5—C4	0.1 (13)
C10—C1—C6—C7	0.5 (11)	C7—C6—C5—C4	177.9 (9)
C2—C1—C10—C9	177.7 (8)	N1—C11—C12—N2	78.7 (10)
C6—C1—C10—C9	−2.9 (12)	C10—C9—C8—C7	−1.4 (14)
C2—C1—C10—N1	2.1 (12)	C6—C5—C4—C3	0.4 (15)
C6—C1—C10—N1	−178.6 (7)	C9—C8—C7—C6	−1.0 (13)
C11—N1—C10—C9	−100.8 (9)	C5—C6—C7—C8	−176.5 (8)
C11—N1—C10—C1	75.0 (10)	C1—C6—C7—C8	1.3 (12)
C6—C1—C2—C3	3.7 (12)	C5—C4—C3—C2	1.2 (15)
C10—C1—C2—C3	−177.0 (8)	C1—C2—C3—C4	−3.2 (13)
C10—N1—C11—C12	179.9 (7)		

Symmetry codes: (i) −*x*+1, −*y*+1, −*z*+1.

### Hydrogen-bond geometry (Å, °)

**Table:**

*D*—H···*A*	*D*—H	H···*A*	*D*···*A*	*D*—H···*A*
N1—H1A···Br1	0.90	2.48	3.364 (8)	168
N1—H1B···Br3^ii^	0.90	2.89	3.618 (7)	139
N2—H2B···Br2^iii^	0.89	2.59	3.339 (9)	143

Symmetry codes: (ii) −*x*+1, −*y*+2, −*z*+1; (iii) −*x*+2, −*y*+1, −*z*+1.
